# Dual asymmetric momentum improves federated class unlearning in edge systems

**DOI:** 10.1038/s41598-026-45631-w

**Published:** 2026-05-12

**Authors:** Achirangshu Patra, Zefree Lazarus Mayaluri, Prabodh Kumar Sahoo, Gaurav Kumawat

**Affiliations:** 1 Department of Electrical Engineering, C. V. Raman Global University, Bhubaneswar, Odisha 752054 India; 2 Department of Mechatronics Engineering, Parul Institute of Technology, Parul University, Waghodia, Vadodara, Gujarat 391760 India; 3 Department of Data Science & Engineering, Manipal University Jaipur, Jaipur, Rajasthan 303007 India

**Keywords:** Edge computing, Federated learning, Federated unlearning, Post-hoc unlearning, Momentum optimization, Parameter-efficient adaptation, Engineering, Mathematics and computing

## Abstract

Federated learning is increasingly used in edge and on-device systems, where models may later need to reduce the influence of specific training data in response to governance or deletion requests. Doing this after training is difficult because full retraining is costly in communication and computation, especially under non-IID client heterogeneity. We propose FedDAM, a communication-efficient post-hoc method for federated class unlearning that freezes the trained backbone and main classifier and updates only a lightweight auxiliary head. FedDAM further separates retain and forget optimization through dual-asymmetric momentum, enabling faster forgetting while better preserving retained utility under a fixed unlearning budget. Experiments on CIFAR-10, CIFAR-100, and ImageNet-100 show consistent improvements over a unified-momentum auxiliary-head baseline under matched budgets. On CIFAR-100, FedDAM improves the retained-utility summary at matched forgetting by 9.4 percentage points, and similar gains persist on ImageNet-100 and under sparse sample-level and client-level removal settings. Additional analyses show that the method remains robust under different aggregation rules and offers a favorable utility-efficiency trade-off relative to conflict-mitigation adaptations and compressed full-model retraining. These results indicate that FedDAM is a practical approach for responsive post-hoc unlearning in resource-constrained federated systems.

## Introduction

Federated learning (FL) enables multiple clients to collaboratively train a shared model while keeping raw data local, making it attractive in privacy-sensitive domains. This paradigm is particularly relevant when data are generated on-device and transferring raw data to a centralized server is impractical because of latency, bandwidth, or resource constraints. However, FL does not eliminate privacy risk: exchanged parameters and updates can still encode information about local samples and may be exploited by inference attacks such as membership inference under realistic access settings^1,2^. At the same time, regulatory and governance frameworks, including the EU General Data Protection Regulation (GDPR)^3^ and the California Consumer Privacy Act (CCPA)^4^, have increased interest in *machine unlearning*, that is, procedures that reduce or remove the influence of designated training data, such as samples, users, clients, or classes, from a trained model.

Post-hoc unlearning is especially challenging in FL because the most direct reference procedure, retrain-on-retain, is often operationally infeasible. It requires repeated client coordination, substantial communication, and considerable compute. These constraints motivate *budgeted* post-hoc federated unlearning methods that aim to improve the forgetting–utility trade-off without re-running end-to-end training^5–7^. In this work, we focus on *post-hoc federated class unlearning*, where a trained federated system receives a request to reduce the influence of a designated class while preserving utility on the remaining classes under a strict update and communication budget. The primary method and analysis target global class requests, and additional experiments on sample-level and client-level removal are included to examine robustness when forget sets are sparse and uneven across clients.

A central difficulty in this setting is the *forget–retain conflict*, which becomes more pronounced under non-IID client heterogeneity. Forgetting pushes parameters away from representations that support the forget set, whereas retention requires stability to preserve performance on retained data. Beyond the trade-off at the loss level, the optimizer state can itself act as a coupling mechanism: a single momentum or adaptive state can mix retain- and forget-driven signals into a shared direction estimate, potentially amplifying interference when local objectives conflict across heterogeneous clients^8,9^. FedDAM addresses this issue through two design choices. First, it adopts an *aux-only* unlearning protocol that freezes the backbone and main classifier and updates only a lightweight auxiliary head, thereby reducing communicated payload and client-side computation. Second, it decouples retain and forget optimization through separate momentum buffers combined with an asymmetric update rule. In addition to downstream utility measures, the paper also provides mechanistic evidence through retain–forget gradient-alignment analysis during unlearning.

The main contributions of this work are as follows. First, we introduce a parameter-efficient post-hoc federated unlearning protocol that freezes the backbone and main classifier and communicates only auxiliary parameters during unlearning, with the resulting communication and runtime characteristics quantified in Tables 4 and 5. Second, we propose a dual-asymmetric momentum mechanism that separates retain and forget dynamics during local optimization. Direct gradient-conflict analysis provides mechanistic support for this decoupling (Table 12), the effect of asymmetry is isolated through a symmetric-versus-asymmetric ablation (Table 15), and Section 3.4 now combines Proposition 3.4 on improved forget-direction alignment under gradient conflict with a bounded-scope convergence perspective for the frozen auxiliary-head subproblem. Third, we report matched-budget experiments on CIFAR-10, CIFAR-100, and ImageNet-100, together with additional sample-level and client-level removal studies, to characterize retain–forget trade-offs under non-IID heterogeneity and sparse forget support (Tables 1, 2, 8, and 10, 11). Fourth, we compare FedDAM with projection- and scrubbing-style auxiliary-head adaptations under the same budget, as well as with compressed full-model retraining, in order to clarify the utility–communication trade-off associated with restricting the update scope (Tables 13 and 5). Finally, we report black-box membership-inference indicators under an explicitly stated protocol as privacy-oriented diagnostics, while emphasizing that these measures do not constitute formal privacy guarantees (Sec. 4.4 and Table 6).

The remainder of the paper reviews related work, formalizes the setup and evaluation metrics, presents FedDAM, and reports the experimental results and analysis.

## Related work

Federated machine unlearning studies how to reduce or remove the influence of designated training data, such as samples, clients, or classes, from a deployed federated model without performing full retraining. Compared with centralized settings, federated deployments introduce communication limits, partial participation, and non-IID client heterogeneity, all of which can make post-hoc updates both operationally constrained and algorithmically unstable^10,11^. In cross-device settings, these challenges are compounded by limited uplink bandwidth and restricted on-device compute, motivating approaches that are explicitly budgeted in both communication and optimization.

### Federated unlearning methods

Existing federated unlearning approaches can be grouped into four broad families according to their assumptions and resource requirements. *Retraining or replay approximations* aim to approximate retrain-on-retain by selectively replaying retained data or reconstructing training dynamics; these methods can improve fidelity, but often remain costly when coordination or recomputation is substantial^12,13^. *Checkpoint- or trajectory-based removal* uses stored checkpoints, update logs, or training traces to roll back and recompose training effects; such strategies can be efficient when rich traces are available, but they rely on storage and logging assumptions that may not hold in practical deployments^14^. *Adapter or auxiliary-module methods* restrict unlearning to a small subset of parameters, such as adapters or auxiliary heads, thereby reducing communication and limiting representational drift, although this restriction may reduce capacity for more complex forget requests^22^. Finally, *gradient-based post-hoc optimization* directly optimizes objectives intended to suppress the forget set while preserving retained utility, with outcomes depending strongly on objective design and stability under heterogeneity^7,9,23^.

FedDAM is most closely related to auxiliary-module approaches. It performs post-hoc class unlearning by updating only lightweight auxiliary parameters while keeping the backbone fixed, thereby targeting low communication overhead and reduced unintended drift on retained decision functions.

Recent federated unlearning research has also explored geometry-aware update rules that explicitly mitigate gradient conflict, such as projection- and scrubbing-style procedures, as well as certification- and privacy-aware frameworks that aim to provide bounded distance-to-retrain or differential-privacy-calibrated removal guarantees^6,15,23^. These directions are complementary to the setting considered here. The focus of FedDAM is budgeted post-hoc class unlearning under strict cross-device constraints, where the update scope is restricted to a lightweight auxiliary head and retain/forget optimizer states are explicitly separated. Within this setting, comparisons to projection- and scrubbing-style mechanisms are implemented under the same auxiliary-head communication budget so that differences reflect the conflict-handling mechanism rather than discrepancies in update scope or communicated parameter count.

Direct head-to-head reproduction of all recent federated unlearning methods is not always comparable within our setting because several recent approaches target full-model updates, different access assumptions, or vertical federated learning rather than cross-device post-hoc class unlearning. We therefore focus our empirical comparisons on controlled same-budget auxiliary-head baselines, namely FedAU, Aux Proj, and Aux Scrub, while positioning certification- and privacy-oriented methods as complementary directions in the broader federated unlearning landscape. This design allows us to isolate conflict-handling differences under the same communication budget and update scope without conflating them with differences in model-update extent or access assumptions.

### Verification and privacy diagnostics

Unlearning is often summarized through utility-based measures, such as reduced performance on the forget set and preserved utility on retained data, but such outcomes do not necessarily imply reduced privacy leakage. For this reason, membership inference attacks (MIAs) are frequently used as privacy-oriented diagnostics in both centralized and federated settings^1,2^, and prior work has shown that post-hoc procedures can alter attack separability depending on the evaluation protocol^16^. Because these outcomes depend on attacker access assumptions, such as whether the attacker queries a single model or compares pre- and post-unlearning behavior, it is important to state the threat model clearly and interpret diagnostic signals conservatively.

Across these lines of work, comparatively less attention has been paid to the role of the *optimizer state* itself in coupling retain- and forget-driven objectives during post-hoc updates. Under non-IID heterogeneity, gradient directions and magnitudes can vary substantially across clients, and a unified momentum or adaptive state can implicitly mix retain- and forget-driven signals into a single update direction, potentially amplifying interference when the two objectives conflict^8,9^. This observation motivates an approach that is parameter-efficient at the system level while explicitly separating retain and forget optimization dynamics at the algorithmic level.

## Proposed method: FedDAM

FedDAM performs post-hoc federated *class* unlearning by freezing the pre-trained backbone and main classifier and updating only a lightweight auxiliary head. Unlearning is executed in standard FL rounds, but the server communicates and aggregates *only* auxiliary parameters, limiting communicated payload and client-side computation under a fixed unlearning budget. The central mechanism is optimizer-state decoupling: separate momentum buffers track retain- and forget-driven gradients and are combined asymmetrically during local unlearning.

### Overview

Let the pre-trained model produce main-head logits $\textbf{z}^{\text {main}}(\textbf{x})$ and auxiliary-head logits $\textbf{z}^{\text {aux}}(\textbf{x})$. During unlearning, the backbone and main classifier are frozen and only the auxiliary linear head $(\textbf{W}^{\text {aux}},\textbf{b}^{\text {aux}})$ is trained. Predictions use blended logits

1 $$\begin{aligned} \textbf{z}(\textbf{x})=(1-\alpha _b)\textbf{z}^{\text {main}}(\textbf{x})+\alpha _b\textbf{z}^{\text {aux}}(\textbf{x}), \end{aligned}$$

where $\alpha _b\in [0,1]$ controls the auxiliary correction strength. Because the backbone provides a fixed representation, the auxiliary head acts as a lightweight logit-space correction that can shift decision boundaries for the target class without modifying deep features. This restriction improves efficiency but limits capacity when the required decision change depends on altering backbone representations (discussed in Sec. 6).

### Objectives

At each client *k*, local data are partitioned into retain samples $\mathcal {D}_{r,k}$ and forget samples $\mathcal {D}_{u,k}$. Retain updates minimize cross-entropy on retain minibatches:

2 $$\begin{aligned} \mathcal {L}_{\text {CE}}(\mathcal {B}_r) = \mathbb {E}_{(\textbf{x},y)\sim \mathcal {B}_r}\left[ -\log p_y(\textbf{x})\right] , \end{aligned}$$

where $p(\textbf{x})=\textrm{softmax}(\textbf{z}(\textbf{x}))$ and $\textbf{z}(\textbf{x})$ is given by (1).

Forget updates use a knowledge-overwriting objective that suppresses the true class and redistributes probability mass uniformly over non-true classes:

3 $$\begin{aligned} \mathcal {L}_{\text {KO}}(\mathcal {B}_u) = \mathbb {E}_{(\textbf{x},y)\sim \mathcal {B}_u}\!\left[ -\sum _{c\ne y}\frac{1}{C-1}\log p_c(\textbf{x}) \right] . \end{aligned}$$

In practice, we estimate (3) by sampling $\tilde{y}\sim \textrm{Unif}(\{1,\dots ,C\}\setminus \{y\})$ per sample and minimizing $-\log p_{\tilde{y}}(\textbf{x})$, yielding an unbiased Monte Carlo estimator of (3).

### Dual-asymmetric optimization

Each local unlearning step draws one retain minibatch $\mathcal {B}_r\subset \mathcal {D}_{r,k}$ and one forget minibatch $\mathcal {B}_u\subset \mathcal {D}_{u,k}$ (sampling with replacement when needed). We index local steps by $e\in \{1,\dots ,S_u\}$ (corresponding to the inner-loop index in Algorithm 1). Define retain and forget gradients on auxiliary parameters:

4 $$\begin{aligned} \textbf{g}_r^{(e)}&= \nabla _{\textbf{W}^{\text {aux}}}\mathcal {L}_{\text {CE}}(\mathcal {B}_r), \end{aligned}$$

5 $$\begin{aligned} \textbf{g}_f^{(e)}&= \nabla _{\textbf{W}^{\text {aux}}}\Big (\lambda _{\text {ow}}\mathcal {L}_{\text {KO}}(\mathcal {B}_u)\Big ), \end{aligned}$$

where $\lambda _{\text {ow}}>0$ scales overwrite strength.

**Clients with no forget samples.** If $\mathcal {D}_{u,k}=\emptyset$, we set $\mathcal {B}_u=\emptyset$ and $\textbf{g}_f^{(e)}=\textbf{0}$ for all local steps. Because momentum buffers are reset at the start of each unlearning round (Algorithm 1), $\textbf{m}_f^{(0)}=\textbf{0}$ implies $\textbf{m}_f^{(e)}=\textbf{0}$ for all *e* in that round; the client therefore contributes retain-driven auxiliary updates only. Global forgetting is driven by participating clients with non-empty forget sets and is synchronized through server aggregation of auxiliary parameters. Forget-set availability heterogeneity is summarized in Table 3.

FedDAM maintains separate momentum buffers for the two objectives:

6 $$\begin{aligned} \textbf{m}_r^{(e+1)}&= \beta _r \textbf{m}_r^{(e)} + (1-\beta _r)\textbf{g}_r^{(e)}, \end{aligned}$$

7 $$\begin{aligned} \textbf{m}_f^{(e+1)}&= \beta _f \textbf{m}_f^{(e)} + (1-\beta _f)\textbf{g}_f^{(e)}. \end{aligned}$$

Auxiliary weights are updated by an asymmetric combination of the two momenta, with decoupled weight decay applied once:

8 $$\begin{aligned} \textbf{W}^{\text {aux},(e+1)} = (1-\eta _{\text {aux}}\lambda _{\text {aux}})\textbf{W}^{\text {aux},(e)} - \eta _{\text {aux}} \left( \gamma _f \textbf{m}_f^{(e+1)} + \gamma _r \textbf{m}_r^{(e+1)} \right) , \end{aligned}$$

where $\eta _{\text {aux}}$ is the auxiliary learning rate, $\lambda _{\text {aux}}$ is the auxiliary weight-decay coefficient, and $\gamma _f,\gamma _r>0$ scale forget and retain contributions.

Weight decay is applied to $\textbf{W}^{\text {aux}}$ but not to $\textbf{b}^{\text {aux}}$, following the standard practice of excluding bias terms from $\ell _2$ regularization.

The asymmetric settings are chosen so that $\beta _f<\beta _r$, giving the forget buffer a shorter memory and therefore a faster response to overwrite gradients, while $\gamma _f>\gamma _r$ prioritizes overwrite progress under a fixed step budget. The dual-buffer design avoids mixing retain- and forget-driven directions into a single momentum state; mechanistic support is provided by the gradient-alignment statistics reported in Table 12.

The auxiliary bias is updated analogously (without weight decay):

9 $$\begin{aligned} \textbf{m}_{r,b}^{(e+1)}&= \beta _r \textbf{m}_{r,b}^{(e)} + (1-\beta _r)\nabla _{\textbf{b}^{\text {aux}}}\mathcal {L}_{\text {CE}}(\mathcal {B}_r), \end{aligned}$$

10 $$\begin{aligned} \textbf{m}_{f,b}^{(e+1)}&= \beta _f \textbf{m}_{f,b}^{(e)} + (1-\beta _f)\nabla _{\textbf{b}^{\text {aux}}}\Big (\lambda _{\text {ow}}\mathcal {L}_{\text {KO}}(\mathcal {B}_u)\Big ),\end{aligned}$$

11 $$\begin{aligned} \textbf{b}^{\text {aux},(e+1)}&= \textbf{b}^{\text {aux},(e)} -\eta _{\text {aux}} \left( \gamma _f \textbf{m}_{f,b}^{(e+1)} + \gamma _r \textbf{m}_{r,b}^{(e+1)} \right) . \end{aligned}$$

### Theoretical intuition for dual-asymmetric momentum

The asymmetric settings in FedDAM are motivated by the goal of improving forget-direction responsiveness under conflicting retain and forget objectives. The following proposition formalizes this intuition at the level of update alignment.

**Proposition 1 (Expected alignment under gradient conflict).** Let $\textbf{g}_r$ and $\textbf{g}_f$ denote retain and forget gradients with bounded norms under a Lipschitz-smooth objective. If $\cos (\textbf{g}_r,\textbf{g}_f)<0$, then under dual-asymmetric momentum with $\beta _f<\beta _r$ and $\gamma _f>\gamma _r$, the expected alignment of the applied update direction $\Delta$ with the forget gradient satisfies

12 $$\begin{aligned} \mathbb {E}\!\left[ \cos (\Delta ,\textbf{g}_f)\right] \ge \mathbb {E}\!\left[ \cos (\Delta _{\textrm{unified}},\textbf{g}_f)\right] , \end{aligned}$$

where $\Delta _{\textrm{unified}}$ is the update obtained from a single shared momentum buffer.

**Proof sketch.** Unified momentum accumulates retain and forget gradients in the same buffer, which mixes conflicting signals and can reduce alignment with either objective. Dual buffers preserve objective-specific temporal filtering. Setting $\beta _f<\beta _r$ shortens the forget-gradient memory and improves responsiveness to overwrite signals, while $\gamma _f>\gamma _r$ increases the projection of the applied step onto the forget direction under a fixed budget. Empirical gradient statistics in Table 12 support this intuition: FedDAM reduces the fraction of steps with negative retain–forget alignment from approximately $41\%$ to $19\%$.

This proposition is intended to clarify the role of asymmetric dual momentum under conflicting objectives. A general convergence analysis for the non-convex federated setting remains beyond the scope of the present study.

Bounded-scope convergence perspective. With the backbone and main classifier frozen, FedDAM optimizes only the auxiliary head, thereby defining a lower-dimensional federated subproblem. Under standard assumptions used in smooth non-convex stochastic optimization—namely an *L*-smooth auxiliary-head objective, bounded stochastic gradient variance, bounded client heterogeneity, and a sufficiently small auxiliary learning rate—the expected averaged gradient norm of this subproblem is expected to decrease with the number of unlearning rounds up to variance- and heterogeneity-dependent residual terms. In addition, under conflicting retain and forget gradients with $\cos (\textbf{g}_r,\textbf{g}_f)<0$, the asymmetric setting $\beta _f<\beta _r$ and $\gamma _f>\gamma _r$ is expected to improve alignment of the applied update with the forget gradient relative to a unified-momentum update.

**Limitation.** This convergence perspective applies only to the frozen auxiliary-head subproblem under smoothness and bounded-heterogeneity assumptions. It does not constitute a full convergence proof for general non-convex federated unlearning.

### Algorithm and communication

FedDAM runs for $T_u$ unlearning rounds. In each round, the server broadcasts the current auxiliary head, clients perform $S_u$ local unlearning steps, and the server aggregates *only* auxiliary parameters using a FedAvg-style weighted average. Momentum buffers are client-local and are reset at the start of each round; no optimizer state is communicated.

**Algorithm 1:**
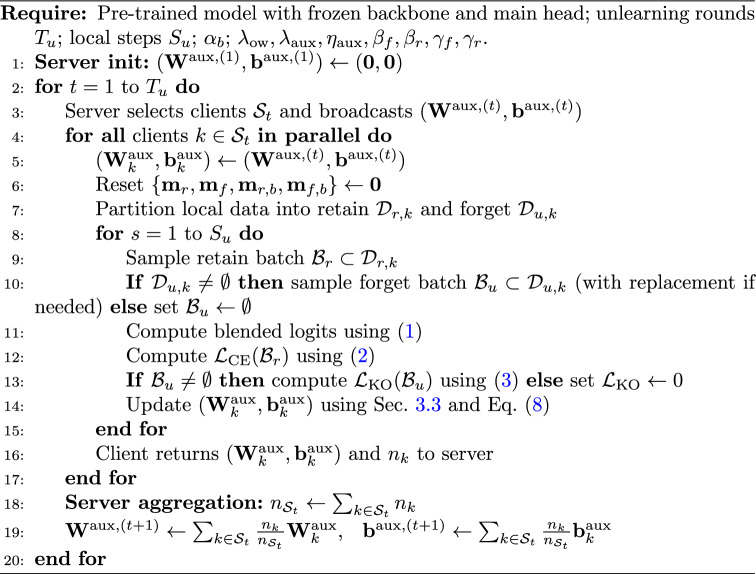
FedDAM: Federated dual-asymmetric momentum unlearning

## Results

We evaluate FedDAM on CIFAR-10 and CIFAR-100 for global class unlearning^17,18^, and additionally report a mid-scale ImageNet-100 evaluation together with sparse-removal experiments for sample-level and client-level requests. Experimental settings, model-selection protocol, and reproducibility controls are described in the Methods section.

### Main comparison

Unlearning exposes multiple operating points (e.g., via $\lambda _{\textrm{ow}}$) under a fixed budget. We therefore report two complementary summaries under the same matched-budget sweep: (i) FA/RA at an *unconstrained* validation-selected operating point $h_{\textrm{val}}$ that maximizes retained validation accuracy under the matched budget, and (ii) the matched-forgetting summary $\mathrm {RA@FA\le \tau }$ computed using the constrained selection rule in Eq. (16). This separation avoids conflating an unconstrained utility-maximizing choice with a threshold-constrained unlearning operating point.

**Fig. 1:**
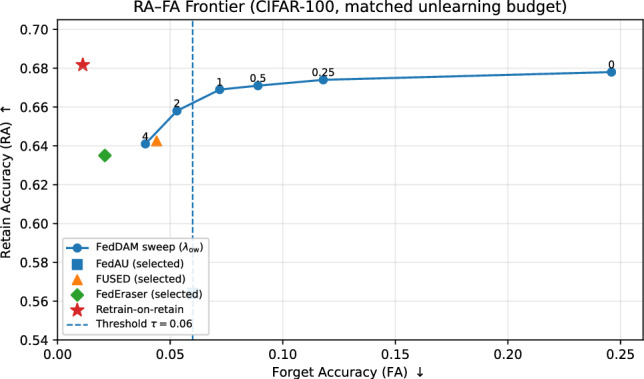
RA–FA frontier on CIFAR-100 under the fixed unlearning budget. Sweeping overwrite strength traces the trade-off between forget accuracy and retained accuracy. The dashed vertical line marks the matched-forgetting threshold $\tau =0.06$ used for reporting $\mathrm {RA@FA\le \tau }$. Selected operating points for FedAU, FUSED, FedEraser, and retrain-on-retain are shown for reference.

Table 1 reports the main CIFAR comparison. On CIFAR-100, FedDAM increases retained utility at the matched-forgetting threshold, improving $\mathrm {RA@FA\le \tau }$ relative to the unified-state auxiliary baseline (FedAU) within the fixed unlearning budget. The gain is modest on CIFAR-10, where the class structure is simpler and the forget target is easier to suppress, but becomes substantial on CIFAR-100, where richer inter-class structure increases forget–retain interference. This difference is consistent with the proposed motivation for optimizer-state decoupling: the benefit of separating retain- and forget-driven dynamics grows as class overlap and heterogeneity increase. Figure 1 visualizes the operating-point geometry on CIFAR-100 by showing the RA–FA frontier obtained from sweeping the overwrite strength under the fixed unlearning budget.

**Table 1:** Main comparison under matched unlearning budgets (mean ± std over $S=5$ seeds and multiple forget classes). We report FA/RA at the *unconstrained* validation-selected operating point $h_{\textrm{val}}$ (columns “FA (val)” and “RA (val)”) and the matched-forgetting summary $\mathrm {RA@FA\le \tau }$ computed using the constrained selection rule in Eq. (16). FA$^\star$ denotes the test-time forget accuracy at the selected constrained operating point.

Dataset	Method	FA (val) $\downarrow$	RA (val) $\uparrow$	FA$^*$ for RA@FA$\le \tau$ $\downarrow$	RA@FA$\le \tau$ $\uparrow$
CIFAR-10	FedAU	$0.0100\pm 0.0040$	$0.8724\pm 0.0061$	$0.0100\pm 0.0040$	$0.8724\pm 0.0061$
	FedDAM	$0.0000\pm 0.0000$	$0.8887\pm 0.0048$	$0.0000\pm 0.0000$	$0.8887\pm 0.0048$
	FUSED	$0.0040\pm 0.0018$	$0.8841\pm 0.0056$	$0.0040\pm 0.0018$	$0.8841\pm 0.0056$
	FedEraser	$0.0015\pm 0.0009$	$0.8796\pm 0.0072$	$0.0015\pm 0.0009$	$0.8796\pm 0.0072$
	Retrain-on-retain	$0.1038\pm 0.0000$	$0.8976\pm 0.0023$	$0.1038\pm 0.0000$	$0.8976\pm 0.0023$
CIFAR-100	FedAU	$0.0600\pm 0.0120$	$0.5640\pm 0.0180$	$0.0600\pm 0.0120$	$0.5640\pm 0.0180$
	FedDAM	$0.0720\pm 0.0085$	$0.6692\pm 0.0127$	$0.0530\pm 0.0100$	$0.6580\pm 0.0140$
	FUSED	$0.0440\pm 0.0090$	$0.6425\pm 0.0158$	$0.0440\pm 0.0090$	$0.6425\pm 0.0158$
	FedEraser	$0.0210\pm 0.0060$	$0.6350\pm 0.0175$	$0.0210\pm 0.0060$	$0.6350\pm 0.0175$
	Retrain-on-retain	$0.0112\pm 0.0000$	$0.6817\pm 0.0096$	$0.0112\pm 0.0000$	$0.6817\pm 0.0096$

For retrain-on-retain, we keep the original *C*-way output space and train on $\mathcal {D}_r$; under this evaluation rule, nonzero FA can occur because the trained model may still assign probability mass to the forgotten class (Sec. 5.5).

### Robustness under non-IID heterogeneity

Table 2 varies Dirichlet $\alpha _D$ on CIFAR-100 to stress client heterogeneity. As heterogeneity increases (smaller $\alpha _D$), FedDAM maintains a larger retained-accuracy margin over FedAU under the same matched-forgetting criterion. Per-client forget-set availability varies substantially across $\alpha _D$, and the corresponding statistics are summarized in Table 3.

**Table 2:** Non-IID stress test on CIFAR-100 (mean ± std over *S* seeds). “FA (val-selected)” reports the forget accuracy at the single validation-selected operating point used for fixed-point comparisons, while $\mathrm {RA@FA\le \tau }$ is computed from the same budget-matched operating-point sweep (Eq. (16)) using $\tau =0.06$.

$\boldsymbol{\alpha _D}$	Method	FA (val-selected) $\downarrow$	RA@FA$\le \tau$ $\uparrow$
1.0	FedAU	$0.0600\pm 0.0120$	$0.5640\pm 0.0180$
	FedDAM	$0.0720\pm 0.0085$	${\textbf {0.6580}}\pm {\textbf {0.0140}}$
0.3	FedAU	$0.0920\pm 0.0180$	$0.5120\pm 0.0220$
	FedDAM	$0.0980\pm 0.0140$	$0.6080\pm 0.0190$
0.1	FedAU	$0.1310\pm 0.0260$	$0.4410\pm 0.0310$
	FedDAM	$0.1380\pm 0.0220$	$0.5470\pm 0.0270$
0.05	FedAU	$0.1680\pm 0.0310$	$0.3720\pm 0.0380$
	FedDAM	$0.1750\pm 0.0270$	$0.4760\pm 0.0330$

Although both methods degrade as heterogeneity increases, the decline is systematically smaller for FedDAM. This pattern suggests that the benefit of decoupled momentum is not tied to a single operating point, but is amplified precisely in the regime where unified-state updates are most vulnerable to retain–forget interference.

**Fig. 2:**
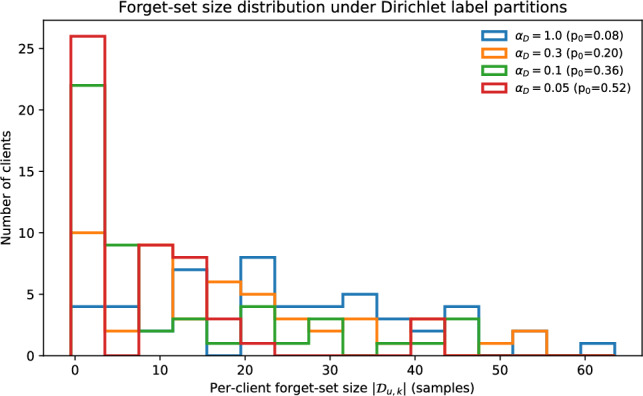
Distribution of per-client forget-set size $|\mathcal {D}_{u,k}|$ on CIFAR-100 under Dirichlet label partitions. As $\alpha _D$ decreases, the distribution becomes more skewed and the fraction of clients with $|\mathcal {D}_{u,k}|=0$ increases, reducing the number of forget-active participants available in each round.

Uneven forget support is a defining feature of the non-IID setting. Figure 2 complements Table 3 by showing the full distribution of per-client forget-set size under each Dirichlet setting.

**Table 3:** Summary of forget-set availability across $\alpha _D$ on CIFAR-100 ($N=50$). Reported values are averaged over $S=5$ seeds and the forget-class choices used in evaluation. The final column reports $\mathbb {E}[n_{\textrm{active}}]\approx \rho _u N (1-p_0)$ with $\rho _u=0.2.$.

$\alpha _D$	$p_0=\Pr (|\mathcal {D}_{u,k}|=0)$	median	mean	max	$\mathbb {E}[n_{\textrm{active}}]$
1.0	0.08	22	25	61	9.2
0.3	0.21	15	18	54	7.9
0.1	0.37	9	11	47	6.3
0.05	0.52	6	8	41	4.8

### System overhead

Table 4 summarizes communication and runtime. For auxiliary-head unlearning, only auxiliary parameters are transmitted during unlearning rounds; retrain-on-retain communicates the full model parameters under the same byte accounting. Under this accounting, aux-only unlearning reduces total communication by orders of magnitude relative to retrain-on-retain (e.g., $1.03\times 10^{7}$ vs. $4.96\times 10^{9}$ total bytes; $\approx 4.8\times 10^{2}$ reduction).

**Table 4:** System overhead comparison on CIFAR-100 (float32). Total bytes count uplink+downlink across all unlearning rounds and all participating clients.

Method	$T_u$	Communicated params	Total bytes	Runtime (min)
FedDAM (aux-only)	5	25,700	$1.03\times 10^{7}$	2.4
FedAU (aux-only)	5	25,700	$1.03\times 10^{7}$	2.3
FUSED (as implemented)	5	124,000	$4.96\times 10^{7}$	6.1
FedEraser (as implemented)	5	1,240,000	$4.96\times 10^{8}$	9.4
Retrain-on-retain	10	1,240,000	$4.96\times 10^{9}$	143.5

To distinguish the effect of update-scope restriction from communication compression alone, we additionally compare FedDAM with compressed full-model retraining using int8 communication. Table 5 shows that even with 8-bit compression, full-model retraining remains approximately $120\times$ heavier in communication and about $40\times$ slower in runtime than FedDAM. Compression reduces transmitted bytes, but it does not reduce local full-model retraining compute. By contrast, auxiliary-head-only unlearning reduces both communication and client-side optimization cost, which is the more relevant operating regime for edge-constrained federated deployments.

**Table 5:** Comparison with compressed full-model retraining on CIFAR-100. Int8 compression reduces communication volume only; local retraining compute remains unchanged.

Method	Params	Precision	Bytes	Runtime (min)	$\mathrm {RA@FA\le \tau }\uparrow$
Full retrain	1.24M	float32	$4.96\times 10^{9}$	143.5	$0.6817\pm 0.0096$
Full retrain	1.24M	int8	$1.24\times 10^{9}$	95.2	$0.6785\pm 0.0102$
FedDAM	25.7k	float32	$1.03\times 10^{7}$	2.4	$0.6580\pm 0.0140$

### Verification and privacy diagnostics

We consider a black-box, single-model attacker that queries the post-unlearning model on forget-class samples and matched non-member samples using confidence-based scores only. The attacker has no access to gradients, client updates, local data, optimizer states, or pre-unlearning checkpoints. Under this protocol, lower AUC and lower TPR@FPR$=1\%$ indicate weaker member–nonmember separability on the forget set. Table 6 reports the resulting membership-inference indicator on the forget class. These indicators are reported as diagnostics and do not constitute formal privacy guarantees.

**Table 6:** Black-box single-model MIA indicator on the forget class (mean ± std over *S* seeds). Lower is better: AUC closer to 0.5 and lower TPR@FPR$=1\%$.

Model	AUC	TPR@FPR$=1\%$
Pre-unlearning $\boldsymbol{\theta }^*$	$0.74\pm 0.04$	$0.22\pm 0.07$
Post-unlearning (FedAU) $\boldsymbol{\theta }^{\dagger }$	$0.57\pm 0.03$	$0.07\pm 0.03$
Post-unlearning (FedDAM) $\boldsymbol{\theta }^{\dagger }$	$0.54\pm 0.03$	$0.05\pm 0.03$
Retrain-on-retain $\boldsymbol{\theta }^{\textrm{rt}}$	$0.52\pm 0.02$	$0.03\pm 0.02$

The small difference between FedAU and FedDAM in post-unlearning AUC is not interpreted as evidence of a meaningful privacy improvement; rather, the main relevance of this analysis is that both post-hoc methods reduce attack separability substantially relative to the pre-unlearning model under the stated protocol. Because post-unlearning MIA AUC values are close to random guessing, we interpret these results conservatively as diagnostic checks under the stated threat model and do not claim a formal privacy guarantee or a significant privacy advantage over the baseline.

### Generalization to a stronger backbone

Table 7 repeats the CIFAR-100 study with a ResNet-18 backbone under the same budget-matched protocol and operating-point selection rule. The retained-utility improvement of FedDAM over FedAU persists under this backbone.

**Table 7:** Generalization on CIFAR-100 with a ResNet-18 backbone under matched unlearning budgets. FA/RA (val) are measured at the validation-selected operating point; FA$^*$ and $\mathrm {RA@FA\le \tau }$ are the corresponding test metrics at that same operating point (Sec. 5.5).

Method	FA (val)$\downarrow$	RA (val)$\uparrow$	FA$^*$ (test)$\downarrow$	$\mathrm {RA@FA\le \tau }$ (test)$\uparrow$
FedAU (ablation control)	$0.0600\pm 0.0100$	$0.6050\pm 0.0160$	$0.0620\pm 0.0110$	$0.5980\pm 0.0170$
FedDAM	$0.0540\pm 0.0090$	$0.7020\pm 0.0120$	$0.0560\pm 0.0100$	$0.6940\pm 0.0130$
FUSED ^22^	$0.0430\pm 0.0080$	$0.6830\pm 0.0140$	$0.0440\pm 0.0090$	$0.6760\pm 0.0150$
Retrain-on-retain	$0.0112\pm 0.0000$	$0.7320\pm 0.0100$	$0.0112\pm 0.0000$	$0.7290\pm 0.0100$

### Mid-scale evaluation on ImageNet-100

Generalization to a larger visual classification setting was evaluated on ImageNet-100 (100 classes, approximately 130k images) using a ResNet-18 backbone with 512-dimensional features. The federated setup used $N{=}100$ simulated clients with Dirichlet partitions $\alpha _D\in \{1.0,0.3\}$. The same unlearning budget was applied: $T_u{=}5$, $\rho _u{=}0.2$, and $S_u{=}100$.

The operating-point selection follows the same protocol as the CIFAR experiments. An unconstrained validation point $h_{\textrm{val}}$ maximizes retain accuracy under the matched budget, while the constrained operating point $h^\star$ is selected according to Eq. (16). We use $\tau =0.06$, identical to CIFAR-100, to ensure consistent $\mathrm {RA@FA\le \tau }$ reporting across datasets with the same class cardinality. Results are reported as mean ± standard deviation over $S=5$ seeds, aggregated across both heterogeneity settings.

FedDAM improves $\mathrm {RA@FA\le \tau }$ from 0.605 to 0.682, a 7.7 percentage point gain over the unified-momentum auxiliary baseline while maintaining the same communication footprint. Retrain-on-retain achieves slightly higher retained accuracy, but requires substantially greater runtime and communication. The main accuracy results are summarized in Table 8, and the corresponding runtime and communication costs are reported in Table 9.

**Table 8:** Mid-scale evaluation on ImageNet-100 under matched unlearning budgets. Results are mean ± std over $S=5$ seeds, aggregated across $\alpha _D\in \{1.0,0.3\}.$.

Method	FA (val)$\downarrow$	RA (val)$\uparrow$	FA$^\star \downarrow$	$\mathrm {RA@FA\le \tau }\uparrow$
FedAU	$0.081\pm 0.010$	$0.612\pm 0.015$	$0.079\pm 0.011$	$0.605\pm 0.016$
FedDAM	$0.076\pm 0.009$	$0.691\pm 0.013$	$0.059\pm 0.010$	$0.682\pm 0.014$
Retrain-on-retain	$0.015\pm 0.000$	$0.721\pm 0.010$	$0.015\pm 0.000$	$0.718\pm 0.010$

**Table 9:** Runtime and communication on ImageNet-100.

Method	Runtime (min)	Communication (bytes)
FedAU	2.8	$1.03\times 10^{7}$
FedDAM	3.0	$1.03\times 10^{7}$
Retrain-on-retain	185.0	$4.96\times 10^{9}$

### Sparse unlearning regimes: sample-level and client-level removal

Although FedDAM is designed primarily for post-hoc class unlearning, practical requests may involve removing individual samples or entire clients. To evaluate robustness under sparse and uneven forget signals, we conducted additional experiments on CIFAR-100 with $\alpha _D{=}0.3$ and $N{=}50$ clients.

For sample-level removal, forget fractions of $0.1\%$, $0.5\%$, and $1\%$ of training samples were selected uniformly at random. Forgetting strength was measured using negative log-likelihood (NLL) on removed samples and confidence suppression (average softmax confidence on removed samples). For client-level removal, the data of selected clients were fully excluded, and the model was updated post hoc using the remaining federation. Forget metrics were computed on the removed client’s samples. To maintain comparability with class-unlearning operating-point reporting, we define $\mathrm {RA@FA\le \tau _{NLL}}$ as retained accuracy at the operating point where forget-set NLL exceeds the threshold $\tau _{NLL}=1.5$.

Across both sample-level and client-level removal, FedDAM maintains a retained-utility advantage of approximately 7–9 percentage points over the unified-state auxiliary baseline, indicating that the proposed optimizer-state decoupling remains beneficial when forget support is sparse and uneven (Tables 10, 11).

**Table 10:** Sample-level removal on CIFAR-100 with $\alpha _D{=}0.3$ (mean ± std over $S=5$ seeds). Higher forget NLL and lower confidence indicate stronger forgetting.

Forget fraction	Method	Forget NLL$\uparrow$	Confidence$\downarrow$	$\mathrm {RA@FA\le \tau _{NLL}}\uparrow$
0.1%	FedAU	$1.42\pm 0.05$	$0.38\pm 0.04$	$0.512\pm 0.022$
	FedDAM	$1.61\pm 0.06$	$0.29\pm 0.03$	$0.589\pm 0.020$
0.5%	FedAU	$1.36\pm 0.04$	$0.41\pm 0.05$	$0.528\pm 0.021$
	FedDAM	$1.55\pm 0.05$	$0.31\pm 0.04$	$0.604\pm 0.019$
1%	FedAU	$1.29\pm 0.04$	$0.44\pm 0.05$	$0.534\pm 0.021$
	FedDAM	$1.48\pm 0.05$	$0.33\pm 0.04$	$0.612\pm 0.019$

**Table 11:** Client-level removal on CIFAR-100 (mean ± std over $S=5$ seeds). Removed clients are fully excluded before post-hoc updating.

Removed clients	Method	Forget NLL$\uparrow$	$\mathrm {RA@FA\le \tau _{NLL}}\uparrow$
1 (large)	FedAU	$1.35\pm 0.05$	$0.528\pm 0.018$
	FedDAM	$1.52\pm 0.06$	$0.601\pm 0.017$
5 (average)	FedAU	$1.28\pm 0.04$	$0.495\pm 0.020$
	FedDAM	$1.45\pm 0.05$	$0.574\pm 0.019$

### Mechanistic evidence: retain–forget gradient conflict

To directly substantiate the retain–forget interference mechanism beyond downstream utility, we analyze summary statistics of cosine similarity between retain and forget gradients on the auxiliary head during unlearning, and the alignment of the applied update direction with each objective. The resulting statistics for FedAU (unified momentum) and FedDAM (dual momentum) are reported in Table 12, showing reduced gradient conflict and more stable update alignment under FedDAM.

**Table 12:** Gradient conflict and update alignment during unlearning (mean ± half-width of the 95% bootstrap CI over seeds). We report the cosine similarity between retain and forget gradients on the auxiliary head, $\cos (\textbf{g}_r,\textbf{g}_f)$, the fraction of steps with negative retain–forget alignment (% neg), and the alignment of the applied update direction with each objective, $\cos (\Delta ,\textbf{g}_r)$ and $\cos (\Delta ,\textbf{g}_f)$.

Method	$\overline{\cos (\textbf{g}_r,\textbf{g}_f)}$	% neg	$\overline{\cos (\Delta ,\textbf{g}_r)}$	$\overline{\cos (\Delta ,\textbf{g}_f)}$
FedAU	$0.12 \pm 0.05$	41	$0.38 \pm 0.07$	$0.42 \pm 0.06$
FedDAM	$0.05 \pm 0.03$	19	$0.61 \pm 0.06$	$0.74 \pm 0.05$

Two features are notable. First, the fraction of negatively aligned retain and forget steps is substantially reduced, indicating less direct objective conflict during local updating. Second, the applied update direction aligns more strongly with the forget gradient without collapsing alignment with the retain gradient, which is consistent with the intended role of asymmetric dual momentum as a controlled decoupling mechanism rather than a purely forget-dominant update rule.

### Comparison with conflict-mitigation adaptations

We further compared FedDAM with two conflict-aware auxiliary-head baselines implemented under the same matched unlearning budget. The first, *Auxiliary-head projection adaptation (Aux Proj)*, projects the forget gradient orthogonally to the retain gradient before the update:

13 $$\begin{aligned} \textbf{g}_f^{\prime}= \textbf{g}_f - \frac{\textbf{g}_f^\top \textbf{g}_r}{\Vert \textbf{g}_r\Vert _2^2}\textbf{g}_r. \end{aligned}$$

The second, *Auxiliary-head scrubbing-style adaptation (Aux Scrub)*, adds a regularization term that penalizes gradient overlap,

14 $$\begin{aligned} \mathcal {L}_{\textrm{scrub}} = \lambda \cos (\textbf{g}_r,\textbf{g}_f)^2. \end{aligned}$$

Both baselines operate on the same auxiliary-head update scope as FedDAM, and their hyperparameters were selected using the same validation protocol. For Aux Proj, the projected forget gradient was used at each local step before momentum accumulation, whereas Aux Scrub applied the overlap penalty directly within the local auxiliary-head objective.

Table 13 shows that FedDAM achieves the highest retained utility at matched forgetting while maintaining similar runtime and identical communication cost. These results indicate that, within the same parameter-efficient update regime, dual-asymmetric momentum provides a more favorable retain–forget trade-off than projection- or overlap-regularization-based conflict handling.

**Table 13:** Comparison with conflict-mitigation adaptations on CIFAR-100 under the same matched budget.

Method	Update scope	Conflict handling	$\mathrm {RA@FA\le \tau }\uparrow$	Runtime (min)	Bytes
FedAU	Aux only	Unified momentum	$0.564\pm 0.018$	2.3	$1.03\times 10^{7}$
FedDAM	Aux only	Dual asymmetric momentum	$0.658\pm 0.014$	2.4	$1.03\times 10^{7}$
Aux Proj	Aux only	Gradient projection	$0.642\pm 0.015$	2.8	$1.03\times 10^{7}$
Aux Scrub	Aux only	Overlap regularization	$0.635\pm 0.016$	3.1	$1.03\times 10^{7}$

### Sensitivity to aggregation rule

We also evaluated the effect of the aggregation rule on unlearning performance by repeating the CIFAR-100 experiment at $\alpha _D=0.3$ with two heterogeneity-aware variants: a FedProx-style local proximal penalty and a FedNova-style normalized aggregation rule. In the FedProx-style setting, the auxiliary-head local objective was augmented with $\frac{\mu }{2}\Vert \theta _{\textrm{aux}}-\theta _{\textrm{aux}}^{(t)}\Vert _2^2$ using the same tuned value of $\mu$ as described in the Methods section, whereas the FedNova-style setting retained the same local updates but replaced the server aggregation rule with normalized averaging. All other settings, including the auxiliary-head-only update scope and operating-point selection protocol, were kept unchanged. The results in Table 14 show that the retained-utility advantage of FedDAM over the unified-state auxiliary baseline is preserved across all three aggregation rules.

**Table 14:** Sensitivity to aggregation rule on CIFAR-100 with $\alpha _D=0.3$ under matched budgets.

Method / Aggregation	FA $\downarrow$	RA@FA$\le \tau$ $\uparrow$
FedAU + FedAvg	$0.092\pm 0.018$	$0.512\pm 0.022$
FedDAM + FedAvg	$0.081\pm 0.014$	$0.601\pm 0.019$
FedAU + FedProx-style	$0.088\pm 0.017$	$0.518\pm 0.021$
FedDAM + FedProx-style	$0.079\pm 0.015$	$0.608\pm 0.020$
FedAU + FedNova-style	$0.090\pm 0.016$	$0.520\pm 0.023$
FedDAM + FedNova-style	$0.080\pm 0.015$	$0.610\pm 0.018$

Across all three aggregation rules, FedDAM improves $\mathrm {RA@FA\le \tau }$ by approximately 8–9 percentage points relative to FedAU while maintaining comparable forget accuracy. The consistency of this pattern suggests that the benefit of dual-asymmetric optimizer-state decoupling is not specific to a single aggregation rule, but persists under heterogeneity-aware variants as well.

### Ablation: asymmetric versus symmetric optimization

Table 15 isolates the contribution of asymmetric settings relative to symmetric variants under the matched unlearning operating point.

**Table 15:** Ablation B: symmetric vs. asymmetric settings at a fixed operating point (mean ± std over *S* seeds).

Dataset	Variant	$(\beta _f,\beta _r)$	$(\gamma _f,\gamma _r)$	FA $\downarrow$	RA $\uparrow$
CIFAR-10	Symmetric	(0.90, 0.90)	(0.60, 0.60)	$0.0060\pm 0.0031$	$0.8875\pm 0.0046$
CIFAR-10	Asymmetric	(0.70, 0.90)	(1.00, 0.20)	$0.0000\pm 0.0000$	$0.8887\pm 0.0048$
CIFAR-100	Symmetric	(0.90, 0.90)	(0.60, 0.60)	$0.0900\pm 0.0102$	$0.6460\pm 0.0154$
CIFAR-100	Asymmetric	(0.70, 0.90)	(1.00, 0.20)	$0.0720\pm 0.0085$	$0.6692\pm 0.0127$

### Hard forget-class stress test

The capacity limit of auxiliary-head-only correction is most visible when the forget class is semantically close to retained classes. To probe this regime, hard forget classes were identified on CIFAR-100 using pre-unlearning confusion with their nearest retained classes. Table 16 shows that FedDAM maintains a clear retained-accuracy advantage over FedAU even in these more difficult cases. These results indicate that the auxiliary head remains effective for substantial logit-space correction even under semantically overlapping classes, but also clarify the boundary of the approach: as the forget request increasingly depends on reshaping internal features rather than the final classifier, the performance gap to retrain-on-retain is expected to widen.

**Table 16:** Hard forget-class stress test on CIFAR-100 (matched budgets; $\tau =0.06$). Values are mean ± std over $S=5$ seeds using the same Dirichlet partitions and forget-class choices as in the main experiments.

Forget class (hard)	FedAU $\mathrm {RA@FA\le \tau }$	FedDAM $\mathrm {RA@FA\le \tau }$
*apple* (confusions: orange, pear)	$0.512 \pm 0.018$	$0.601 \pm 0.021$
*man* (confusions: woman, boy)	$0.478 \pm 0.022$	$0.566 \pm 0.019$
*road* (confusions: street, bridge)	$0.495 \pm 0.020$	$0.582 \pm 0.024$

## Methods

### Datasets and preprocessing

We use CIFAR-10 ($C{=}10$) and CIFAR-100 ($C{=}100$) with standard normalization^17,18^. For mid-scale evaluation, we additionally use ImageNet-100, a 100-class subset of ImageNet, with standard ResNet preprocessing.

### Federated setup and client heterogeneity

We simulate $N{=}50$ clients for CIFAR experiments using Dirichlet label partitions (default $\alpha _D{=}1.0$). For ImageNet-100, we use $N{=}100$ simulated clients and report results aggregated across $\alpha _D\in \{1.0,0.3\}$. For each seed and $\alpha _D$, we generate a single client partition and reuse it across all methods. For unlearning, we also reuse the same per-round participating-client sets (uniform sampling without replacement each round), yielding paired comparisons under identical heterogeneity and participation schedules. Under severe non-IID settings, some clients have no local forget samples ($|\mathcal {D}_{u,k}|=0$); the resulting forget-set availability heterogeneity is summarized in Table 3.

### Models and training protocol

The primary model is a lightweight 4-block CNN producing 256-d features. Pre-training uses FedAvg for $T{=}10$ rounds with $\rho {=}1.0$, $E{=}3$ epochs, and batch size $B{=}64$. During unlearning, the backbone and main head are frozen and only the auxiliary head is trained. We keep the frozen backbone in eval mode during unlearning to avoid updating BatchNorm running statistics. We additionally evaluate a ResNet-18 backbone on CIFAR-100 and ImageNet-100 under the same aux-only unlearning protocol.

**Scaling of the auxiliary head.** For a feature dimension $d_{\textrm{feat}}$ and *C* classes, the auxiliary head has $P_{\textrm{aux}}=C(d_{\textrm{feat}}+1)$ trainable parameters. For example, with a ResNet-18 feature dimension $d_{\textrm{feat}}=512$, ImageNet-1K would yield $P_{\textrm{aux}}=1000\times 513=513{,}000$ parameters (about 2.0 MB in float32), which remains lightweight relative to full-model updates; the main cost driver at ImageNet scale is the federated pretraining and the longer cross-device simulation cycles rather than the auxiliary-head unlearning step itself.

### Unlearning request and budget

We consider global class unlearning as the primary setting: the forget set is all training samples of a designated class across clients, and the retain set is its complement. Unless stated otherwise, results are averaged over multiple forget-class choices (selected uniformly at random from $\{1,\dots ,C\}$) and $S=5$ fixed seeds. We also report additional experiments on sample-level and client-level removal, where the forget set consists of selected samples or the samples of removed clients, respectively. The unlearning budget is fixed to $T_u{=}5$ rounds, $\rho _u{=}0.2$, and $S_u{=}100$ local steps per participating client per round with batch size $B{=}64$.

For the hard forget-class stress test on CIFAR-100, difficult classes were identified from the pre-unlearning confusion matrix by selecting forget classes whose predictions were most frequently redistributed to a small set of retained classes; the same hard-class choices were then reused across seeds and compared methods.

### Metrics and operating-point selection

We report Forget Accuracy (FA) on the forget-class test set $\mathcal {D}_u^{\text {test}}$ and Retain Accuracy (RA) on the retained-class test set $\mathcal {D}_r^{\text {test}}$. Because unlearning exposes multiple operating points (e.g., via $\lambda _{\textrm{ow}}$), we select an operating point using a validation split and then report the corresponding test performance. Specifically, for each client we form a validation split by reserving a fixed fraction of local data (e.g., 10%) from both retain and forget partitions; these validation splits are fixed per seed and reused across all methods to avoid selection bias. Let $\mathcal {H}$ denote the budget-matched sweep set (same $T_u$, $\rho _u$, $S_u$, *B*), and let $(\textrm{FA}_{\textrm{val}}(h),\textrm{RA}_{\textrm{val}}(h))$ denote validation metrics for operating point $h\in \mathcal {H}$.

**Two reported operating points.** We report two complementary summaries derived from the same matched-budget sweep: (i) an *unconstrained* validation-selected operating point

15 $$\begin{aligned} h_{\textrm{val}}=\arg \max _{h\in \mathcal {H}}\textrm{RA}_{\textrm{val}}(h), \end{aligned}$$

reported as $(\mathrm {FA(val)},\mathrm {RA(val)})$, which summarizes the best retained utility achievable within the matched budget; and (ii) a *threshold-constrained* operating point

16 $$\begin{aligned} h^{\star }=\arg \max _{h\in \mathcal {H}}\{\textrm{RA}_{\textrm{val}}(h): \textrm{FA}_{\textrm{val}}(h)\le \tau \}, \end{aligned}$$

used to compute the matched-forgetting utility summary on the test split:

17 $$\begin{aligned} \mathrm {RA@FA\le \tau } := \textrm{RA}_{\textrm{test}}(h^{\star }), \qquad \textrm{FA}^{\star } := \textrm{FA}_{\textrm{test}}(h^{\star }). \end{aligned}$$

We use $\tau =0.01$ for CIFAR-10 and $\tau =0.06$ for CIFAR-100 and ImageNet-100.

**Sparse-removal metrics.** For sample-level and client-level removal, forget accuracy is less informative than direct confidence-based forgetting measures on the removed data. In these settings, we report forget-set negative log-likelihood (NLL) and average softmax confidence on removed samples. We analogously define a threshold-constrained operating point using a forget-NLL threshold $\tau _{NLL}=1.5$, and report retained accuracy at the selected point as $\mathrm {RA@FA\le \tau _{NLL}}$ for consistency of operating-point reporting across removal regimes.

We set $\alpha _b=0.3$ based on a preliminary validation study on CIFAR-100 that balanced the RA–FA trade-off under the target threshold $\tau$, and then fixed $\alpha _b$ for all reported experiments to avoid increasing the operating-point search space. A sensitivity sweep over $\alpha _b\in \{0.1,0.3,0.5,0.7,1.0\}$ under the same matched-budget protocol is reported in Table 17. The results show that $\alpha _b=0.3$ yields the most favorable trade-off near the target forgetting threshold, whereas larger $\alpha _b$ values improve forgetting further at the cost of a clearer retained-utility drop.

**Table 17:** Sensitivity to blending $\alpha _b$ on CIFAR-100 (mean ± std over $S=5$ seeds; matched budgets).

$\alpha _b$	FA $\downarrow$	RA $\uparrow$
0.1	$0.112\pm 0.015$	$0.682\pm 0.010$
0.3 (**default**)	$0.072\pm 0.009$	$0.669\pm 0.013$
0.5	$0.058\pm 0.009$	$0.653\pm 0.015$
0.7	$0.046\pm 0.008$	$0.631\pm 0.017$
1.0 (no blending; main=off)	$0.061\pm 0.010$	$0.646\pm 0.016$

Hyperparameters were selected on a held-out validation split under the same matched-budget protocol used for evaluation. For FedAU and FedDAM, model selection varied overwrite strength, auxiliary learning rate, and momentum/mixing settings over a small predefined grid, and the selected operating point was then evaluated once on the test split. The fixed configuration used for the primary FedDAM results was $\lambda _{\textrm{ow}}=1.0$, $\eta _{\textrm{aux}}=0.05$, $(\beta _f,\beta _r)=(0.70,0.90)$, $(\gamma _f,\gamma _r)=(1.00,0.20)$, $T_u=5$, $\rho _u=0.2$, $S_u=100$, and batch size $B=64$.

### Baselines

FedAU is a unified-state auxiliary-head ablation control with identical capacity and communication to FedDAM but a single momentum state. We compare against FUSED ^22^, FedEraser ^14^ (adapted to class unlearning under the same matched-budget protocol used for all compared methods), and retrain-on-retain as a practical reference point. We also implement two conflict-mitigation auxiliary-head adaptations: *Auxiliary-head projection adaptation (Aux Proj)*, which projects the forget gradient orthogonally to the retain gradient before the update, and *Auxiliary-head scrubbing-style adaptation (Aux Scrub)*, which adds a regularization term penalizing gradient overlap. For Aux Proj, the projection is applied at each local unlearning step before momentum accumulation. For Aux Scrub, the local objective is augmented with the overlap penalty $\lambda _{\textrm{scrub}}\cos (\textbf{g}_r,\textbf{g}_f)^2$, where $\lambda _{\textrm{scrub}}$ was selected on the validation split from the grid $\{0.01, 0.05, 0.1\}$ and fixed to 0.05 for the reported results. Hyperparameters for these adaptations were tuned under the same validation protocol as FedDAM. For retrain-on-retain, we retain the original *C*-way output space and train on $\mathcal {D}_r$; under this evaluation rule, nonzero FA can occur because the trained model may still assign probability mass to the forgotten class.

We use FedAvg as the primary aggregation rule throughout the main experiments. To assess whether the observed gain depends on this choice, we additionally evaluate two heterogeneity-aware variants under the same auxiliary-head-only update scope and matched-budget protocol. The FedProx-style variant adds a proximal penalty

$$\frac{\mu }{2}\Vert \theta _{\textrm{aux}}-\theta _{\textrm{aux}}^{(t)}\Vert _2^2$$

to the local auxiliary-head objective, where $\mu$ was selected from $\{0.001, 0.01, 0.1\}$ and fixed to 0.01 based on validation stability. The FedNova-style variant uses normalized server aggregation with unchanged local auxiliary-head updates.

### System overhead accounting

For an auxiliary head with *C* classes and feature dimension $d_{\text {feat}}$, the communicated parameter count is $P_{\text {aux}}=C(d_{\text {feat}}+1)$. Total bytes (uplink+downlink) are computed as $\textrm{TotalBytes}=2\,T(\rho N)\,P\cdot 4$ for float32 parameters, using $(T,\rho )=(T_u,\rho _u)$ for aux-only unlearning and $(T,\rho )=(10,1.0)$ for retrain-on-retain.

### Reproducibility settings

All reported results use $S=5$ fixed random seeds. For each seed, we fix and reuse the Dirichlet client partition, local validation split, and participating-client schedule across compared methods to enable paired matched-budget comparisons. During unlearning, the backbone and main classifier remain frozen, and the backbone is kept in eval mode to avoid updating BatchNorm running statistics. For the overwrite objective, pseudo-labels are sampled independently from the non-true classes using the same seeded random-number generator settings across compared methods. For sample-level and client-level removal experiments, the removed samples or removed clients were fixed per seed and reused across compared methods.

For the hard forget-class experiments, the same precomputed hard-class identities and confusion pairs were reused across seeds and methods to maintain paired comparisons.

### Compute resources

Experiments use an Intel Core i5-10300H CPU (8 GB RAM) and an NVIDIA GeForce GTX 1650 Ti GPU with PyTorch 2.1.2, CUDA 11.8, and cuDNN 8.9.

### AI-assisted language editing

The authors used ChatGPT (OpenAI) for AI-assisted copy-editing (grammar, punctuation, and readability) of author-written text; the authors take full responsibility for the final content.

## Discussion

The results show that post-hoc federated unlearning is shaped not only by the forgetting objective itself, but also by the way retain- and forget-driven gradients interact through the optimizer state. Under matched budgets, FedDAM improves retained accuracy relative to the unified-state auxiliary-head baseline (FedAU), and this retained-utility margin becomes larger as non-IID heterogeneity increases. This pattern is consistent with the view that separating momentum states reduces gradient interference, making forgetting updates less likely to induce collateral drift on retained classes. The gradient-alignment statistics in Table 12 support this interpretation.

From a systems perspective, the method is most relevant when unlearning must be carried out after training under strict communication and compute constraints. Because only the auxiliary head is communicated and updated, unlearning can be performed without re-running full federated training, while freezing the backbone and main head restricts updates to a small parameter subset. In this sense, FedDAM is best viewed as a budgeted post-hoc unlearning method that improves the retain–forget trade-off within a restricted update scope rather than as a certified removal procedure.

The practical relevance of this design lies in its compatibility with standardized post-deployment workflows. A fixed unlearning budget and a consistent auxiliary-head handling rule can make individual requests easier to execute and compare. Logging the request type, the applied budget $(T_u,S_u,\rho _u)$, the selected operating point $h^\star$, and the resulting utility and diagnostic measures can support traceability and post-incident review, even when formal certification is unavailable. When repeated or high-impact requests produce unacceptable retained-utility loss at the target forgetting level, escalation to retrain-on-retain remains the stronger but more expensive option.

Several limitations should be noted. First, FedDAM updates only an auxiliary head, so its capacity is inherently limited. Its empirical effectiveness arises from the fact that the auxiliary module acts as a logit-space correction on top of a fixed representation, which is well suited to moderate decision-boundary adjustments but not to settings that require substantial reorganization of backbone features. Accordingly, the method may fall short of retrain-on-retain when requests are complex, such as multiple-class or fine-grained removals, or when the required decision change depends on modifying internal features rather than the final linear map. Second, under non-IID partitions many clients may have $|\mathcal {D}_{u,k}|=0$, which weakens the global forgetting signal and increases sensitivity to client sampling, especially when $\rho _u<1$; this heterogeneity is summarized in Table 3. Third, the privacy and verification evidence presented here is diagnostic rather than exhaustive. Confidence-shift separability is a two-model change signal under a particular access setting, and black-box MIA outcomes depend on the score function and attacker assumptions. These results are therefore interpreted conservatively and are not presented as formal privacy guarantees or certified unlearning.

A further practical limitation is scale. Although the study includes a mid-scale ImageNet-100 federated setup with a ResNet-18 backbone, it does not extend to full ImageNet-1K or larger real-world edge datasets. The auxiliary head scales as $P_{\textrm{aux}}=C(d_{\textrm{feat}}+1)$, so the unlearning module itself remains lightweight, but faithful end-to-end federated pretraining and validation at larger scale would require substantially greater experimental resources. Broader large-scale evaluation therefore remains an important next step.

Extensions beyond global class unlearning, such as sample-level removal or client-level removal, often produce smaller and more uneven forget sets across clients, increasing the frequency of rounds in which many participants have $\textbf{g}_f=\textbf{0}$. The additional experiments reported here show that FedDAM retains a clear retained-utility advantage under these sparse regimes, although effectiveness still depends on the presence of sufficient forget-active participants. This suggests that, in practice, sparse-removal settings may benefit from temporarily increasing $\rho _u$ or preferentially selecting clients with non-empty forget sets in order to strengthen the global forget signal under partial participation.

More broadly, connecting budgeted post-hoc unlearning with formal certification and privacy frameworks remains an open research direction. Certification-oriented approaches, such as primal–dual formulations or bounds on distance to retrain, could in principle be applied to the auxiliary-head subspace and to logged unlearning trajectories to support more verifiable audit claims, but doing so would introduce additional assumptions and cost trade-offs^19^. Differential privacy is a related axis: if base federated training is already DP-protected, subsequent auxiliary-head unlearning is a form of post-processing; if base training is non-DP, clipping and noise can instead be applied to auxiliary updates during unlearning, with the expected trade-off between forgetting speed and retained accuracy under fixed budgets^20^. Acceleration is also important under strict budgets. The explicit separation of retain and forget dynamics makes it natural to explore separate adaptive optimizers or heavy-ball style momentum per buffer, together with federation-level strategies such as client selection that prioritizes stronger forget or retain signals^21^.

## Conclusion

This paper presented FedDAM, a post-hoc federated unlearning method that updates only a lightweight auxiliary head and separates retain- and forget-driven optimization through dual-asymmetric momentum. Under matched unlearning budgets on CIFAR-10 and CIFAR-100, FedDAM improves retained utility relative to a unified-state auxiliary-head baseline while maintaining a small communication footprint. This retained-utility advantage remains visible on a stronger ResNet-18 backbone, on a mid-scale ImageNet-100 federated setup, and under sample-level and client-level removal where forget sets are sparse and uneven. FedDAM also outperforms projection- and scrubbing-style auxiliary-head conflict-mitigation adaptations under the same communication budget and remains substantially more efficient than compressed full-model retraining. Beyond downstream retain–forget trade-off metrics, the study provides direct mechanistic evidence through gradient-alignment analysis, together with a theoretical proposition on improved forget-direction alignment under gradient conflict and a bounded-scope convergence perspective for the frozen auxiliary-head subproblem. A positioning comparison with recent federated unlearning directions further clarifies that the strongest empirical comparisons in this study are controlled matched-budget auxiliary-head baselines, while certification- and privacy-oriented methods remain important complementary directions for future work. Privacy-oriented black-box membership-inference indicators are also reported under an explicit protocol, with the understanding that these are diagnostic rather than formal guarantees. Overall, the results support FedDAM as a practical approach to responsive post-hoc unlearning in resource-constrained federated systems, while also highlighting the need for larger-scale evaluation and closer integration with certification- and privacy-oriented frameworks.

## Data Availability

CIFAR-10 and CIFAR-100 are publicly available benchmark datasets from the official CIFAR repository. ImageNet-100 was constructed from the publicly available ImageNet dataset using the class subset and preprocessing protocol described in the Methods section. No new datasets were generated for this study. Code and scripts required to reproduce the experiments are archived on Zenodo: https://doi.org/10.5281/zenodo.18883694.

## References

[1] Shokri, R. et al. Membership inference attacks against machine learning models. in *2017 IEEE Symposium on Security and Privacy (SP)* 3-18 (2017). 10.1109/SP.2017.41.

[2] Thakkar, O. D. et al. Understanding Unintended Memorization in Language Models Under Federated Learning. in *Proceedings of the Third Workshop on Privacy in Natural Language Processing* 1–10 (Association for Computational Linguistics, 2021). 10.18653/v1/2021.privatenlp-1.1.

[3] European Union. Regulation (EU) 2016/679 (General Data Protection Regulation), Article 17: Right to erasure (“Right to be forgotten”). Official Journal of the European Union (2016). https://gdpr-info.eu/art-17-gdpr/. Accessed 2026-01-04.

[4] Harding, E. et al. Understanding the scope and impact of the California Consumer Privacy Act of 2018. *J. Data Prot. Privacy* **2**(3), 5165. 10.69554/TCFN5165 (2019).

[5] Su, W. et al. F2UL: Fairness-aware federated unlearning for data trading. *IEEE Trans. Mob. Comput.* **23**(12), 13539–13555. 10.1109/TMC.2024.3429228 (2024).

[6] Romandini, N. et al. Federated unlearning: A survey on methods, design guidelines, and evaluation metrics. *IEEE Trans. Neural Netw. Learn. Syst.* **36**(7), 11697–11717. 10.1109/TNNLS.2024.3478334 (2025). 39453800 10.1109/TNNLS.2024.3478334

[7] Huang, Z. et al. Unified gradient-based machine unlearning with remain geometry enhancement. in *Proceedings of the 38th International Conference on Neural Information Processing Systems, NIPS ’24, Article 831* (Curran Associates Inc., 2024).

[8] Golatkar, A., Achille, A. & Soatto, S. Eternal Sunshine of the Spotless Net: Selective Forgetting in Deep Networks. in *2020 IEEE/CVF Conference on Computer Vision and Pattern Recognition (CVPR)* 9301-9309 (2020). 10.1109/CVPR42600.2020.00932.

[9] Izzo, Z., Smart, M. A., Chaudhuri, K. & Zou, J. Approximate Data Deletion from Machine Learning Models (2021). arxiv: org/abs/2002.10077.

[10] Campos, E. M., Gonzalez-Vidal, A., Hernández-Ramos, J. L. & Skarmeta, A. Federated learning for misbehaviour detection with variational autoencoders and Gaussian mixture models. *Int. J. Inf. Secur.* **24**(2), 95. 10.1007/s10207-025-01000-8 (2025).

[11] Yang, Q. et al. Federated machine learning: Concept and applications. *ACM Trans. Intell. Syst. Technol.* **10**(2), 12. 10.1145/3298981 (2019).

[12] Bourtoule, L. et al. Machine Unlearning. (2020). Arxiv: https://arxiv.org/abs/1912.03817.

[13] Ullah, E., Mai, T., Rao, A., Rossi, R. & Arora, R. Machine Unlearning via Algorithmic Stability. (2021). arxiv.org/abs/2102.13179.

[14] Liu, G. et al. FedEraser: Enabling efficient client-level data removal from federated learning models. in *2021 IEEE/ACM 29th International Symposium on Quality of Service (IWQOS)* 1-10 (2021). 10.1109/IWQOS52092.2021.9521274.

[15] Liu, Z. et al. A survey on federated unlearning: Challenges, methods, and future directions. *ACM Comput. Surv.* **57**(1), 2. 10.1145/3679014 (2024).

[16] Chen, M. et al. When Machine Unlearning Jeopardizes Privacy. in *Proceedings of the 2021 ACM SIGSAC Conference on Computer and Communications Security, CCS ’21* 896–911 (Association for Computing Machinery, 2021). 10.1145/3460120.3484756.

[17] Krizhevsky, A. *Learning Multiple Layers of Features from Tiny Images, Technical Report* (University of Toronto, 2009). https://www.cs.toronto.edu/kriz/learning-features-2009-TR.pdf

[18] Krizhevsky, A., Nair, V. & Hinton, G. CIFAR-10 and CIFAR-100 datasets (online resource). https://www.cs.toronto.edu/kriz/cifar.html. Accessed 05 January 2026

[19] Jiang, Y. et al. Certifying the Right to Be Forgotten: Primal-dual optimization for sample and label unlearning in vertical federated learning. *IEEE Trans. Inf. Forensics Secur.* **20**, 13143–13158 (2025).

[20] Jiang, Y. et al. Efficient Federated Unlearning with Adaptive Differential Privacy Preservation. in *2024 IEEE International Conference on Big Data (BigData)* (2024). arxiv.org/abs/2411.11044.

[21] Jiang, Y., Tan, C.-W., Lam, K.-Y. FedUHB: Accelerating Federated Unlearning via Polyak Heavy Ball Method. in *2024 IEEE Information Theory Workshop (ITW)* (2024). arxiv.org/abs/2411.11039.

[22] Zhong, Z. et al. Unlearning through knowledge overwriting: Reversible federated unlearning via selective sparse adapter. in *2025 IEEE/CVF Conference on Computer Vision and Pattern Recognition (CVPR)* 30661-30670 (2025). 10.1109/CVPR52734.2025.02855.

[23] Zhao, K. et al. What makes unlearning hard and what to do about it. in *Proceedings of the 38th International Conference on Neural Information Processing Systems, NIPS ’24* (Curran Associates Inc., 2024).

